# Supplementary figures and images for: Bidirectional correlation between gastroesophageal reflux disease and sleep problems: a systematic review and meta-analysis
Source: PeerJ. 2024 Apr 16;12:e17202. doi: 10.7717/peerj.17202 (PMC11027907; doi:10.7717/peerj.17202)

Meta-analysis random-effects estimates (exponential form)  
Study ommited

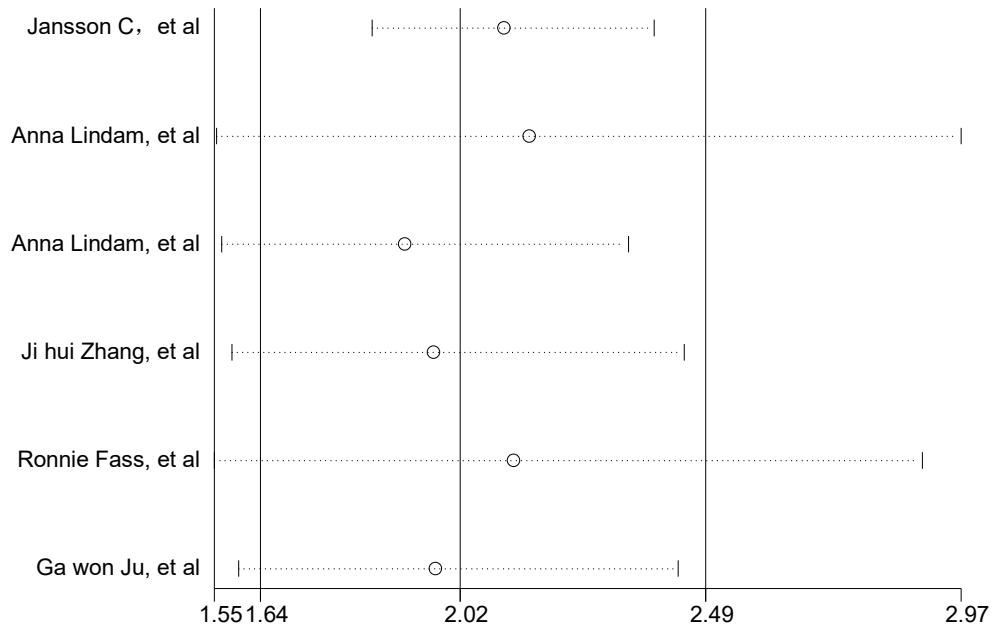

Supplement: Supplemental Information 1 [file peerj-12-17202-s001.pdf]

Meta-analysis random-effects estimates (exponential form)  
Study omitted

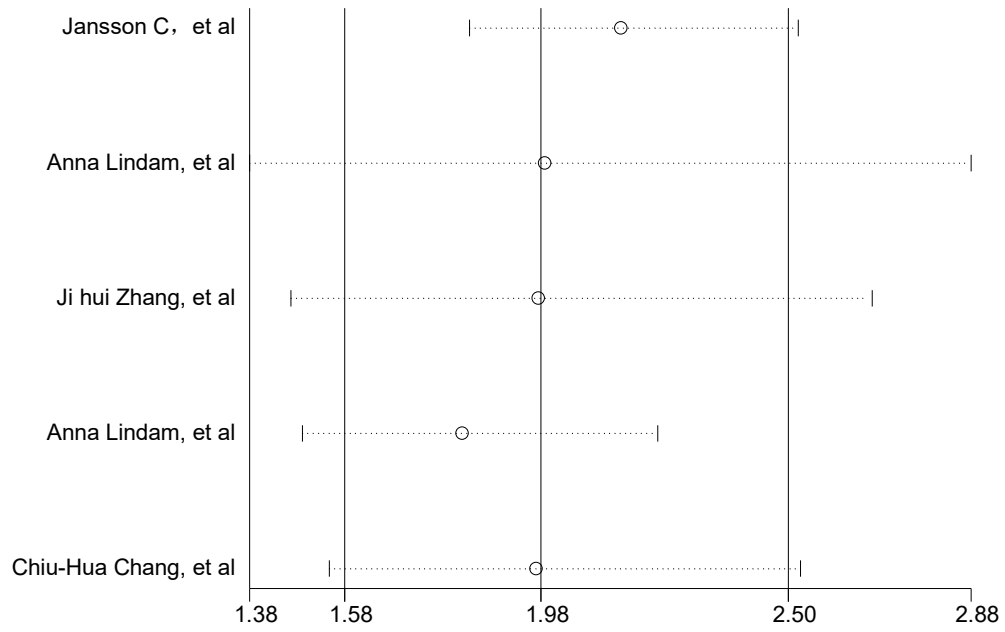

Supplement: Supplemental Information 2 [file peerj-12-17202-s002.pdf]

Meta-analysis random-effects estimates (exponential form)  
Study omitted

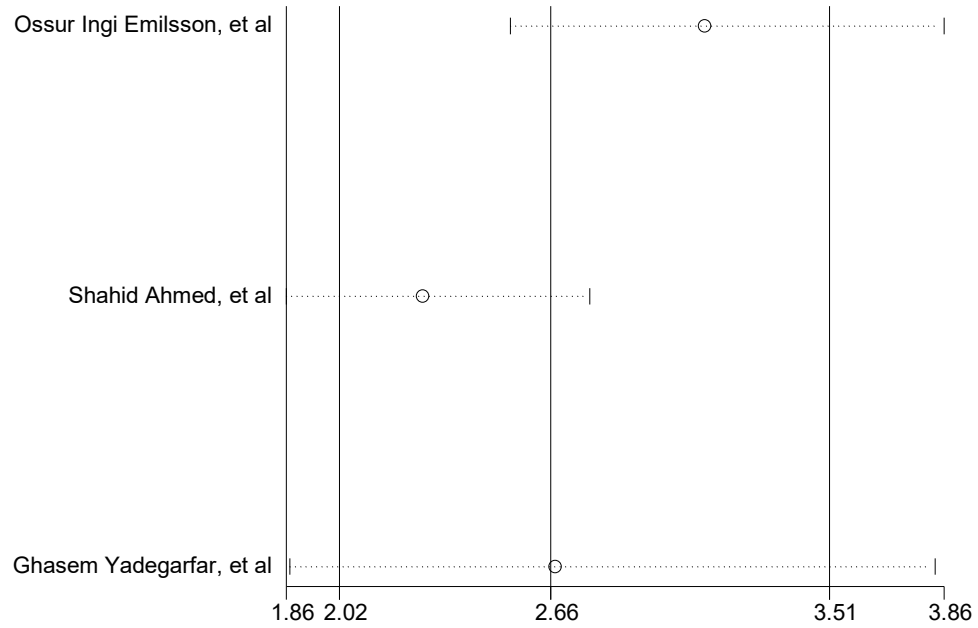

Supplement: Supplemental Information 3 [file peerj-12-17202-s003.pdf]

Meta-analysis random-effects estimates (exponential form)  
Study omitted

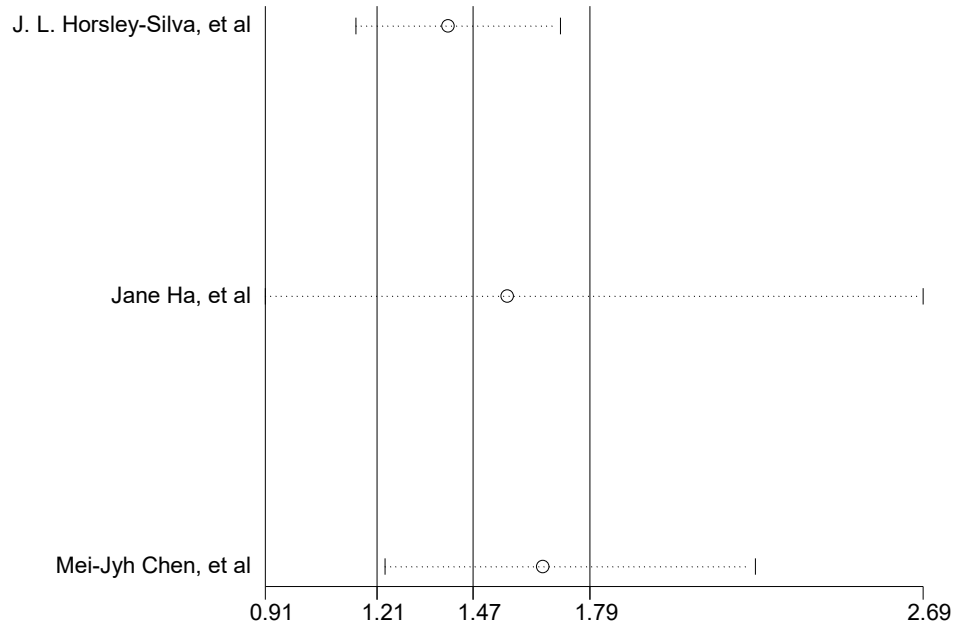

Supplement: Supplemental Information 4 [file peerj-12-17202-s004.pdf]

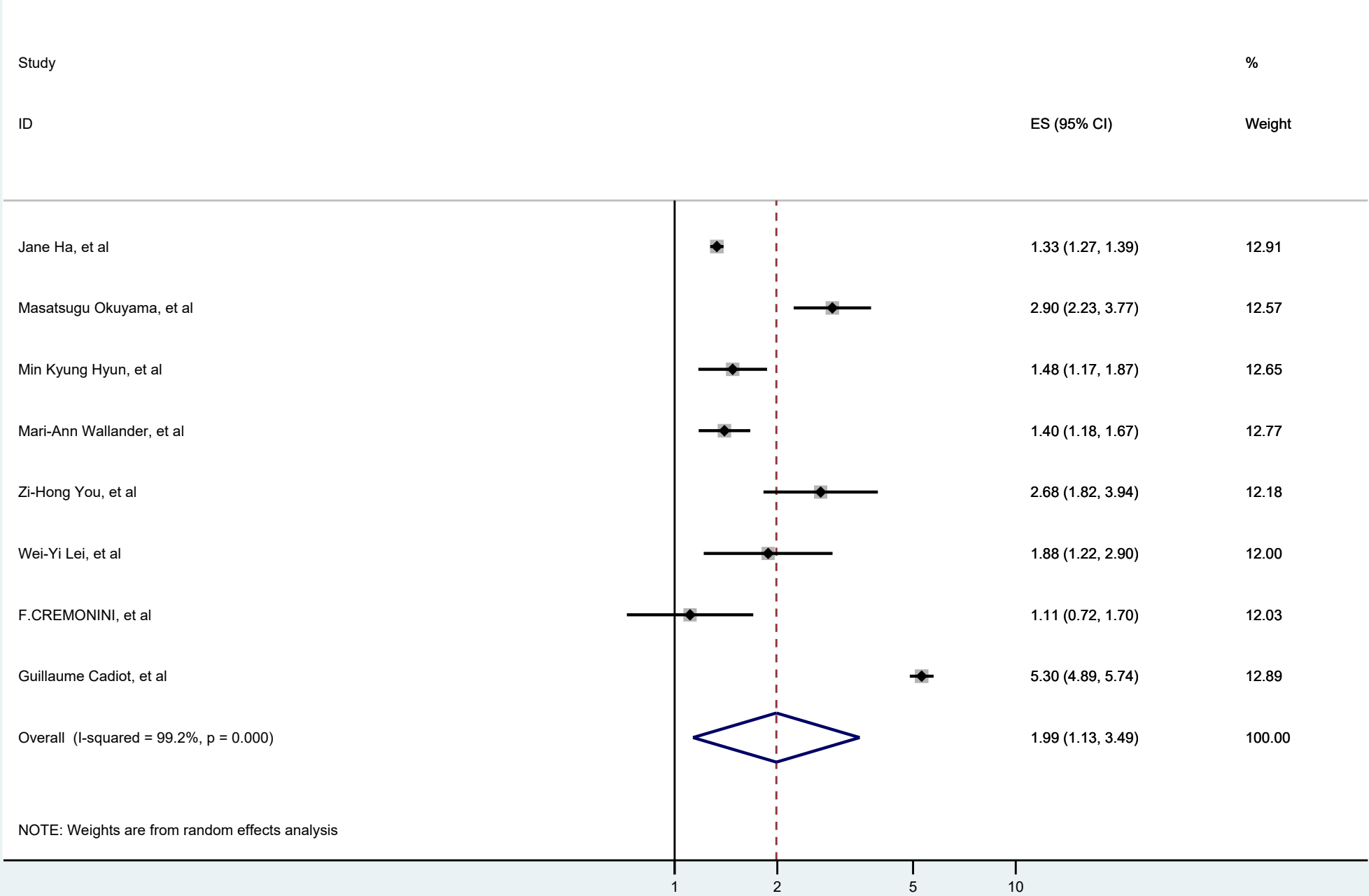

Supplement: Supplemental Information 5 [file peerj-12-17202-s005.pdf]

Meta-analysis random-effects estimates (exponential form)  
Study omitted

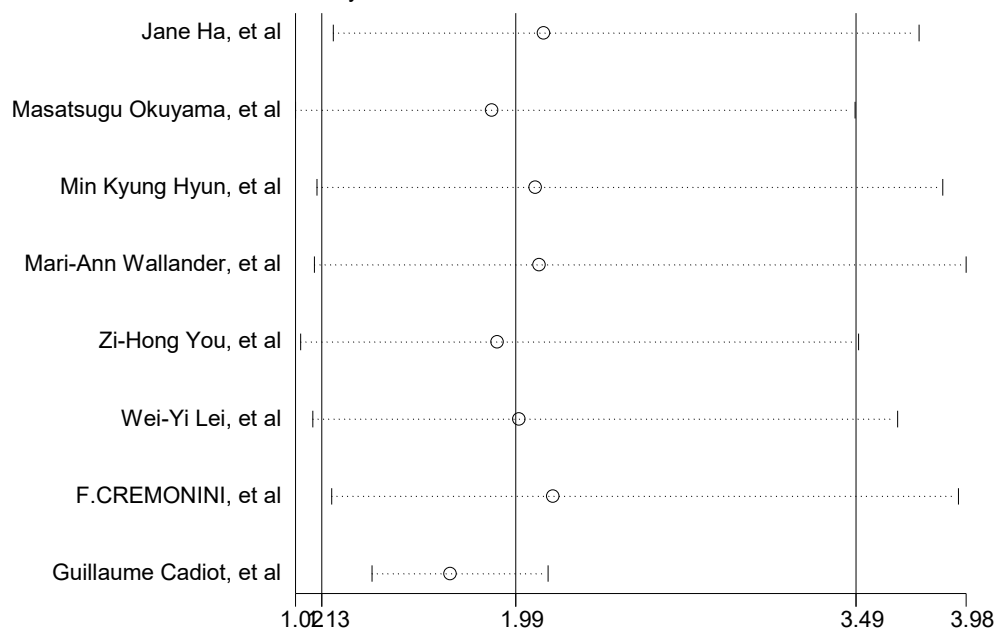

Supplement: Supplemental Information 6 [file peerj-12-17202-s006.pdf]

Meta-analysis random-effects estimates (exponential form)  
Study omitted

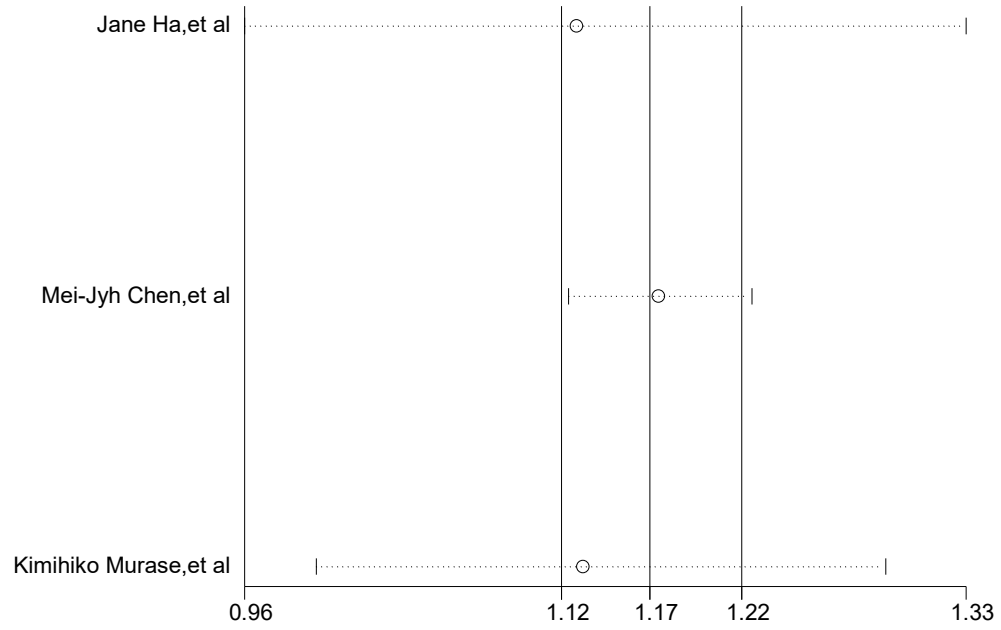

Supplement: Supplemental Information 7 [file peerj-12-17202-s007.pdf]
