# Supplementary material for: Bidirectional correlation between gastroesophageal reflux disease and sleep problems: a systematic review and meta-analysis
Source: PeerJ. 2024 Apr 16;12:e17202. doi: 10.7717/peerj.17202 (PMC11027907; doi:10.7717/peerj.17202)
Supplement: Supplemental Information 8 [file peerj-12-17202-s008.docx]

| **Section and Topic** | **Item #** | **Checklist item** | **Location where item is reported** |
| --- | --- | --- | --- |
| **TITLE** | | |  |
| Title | 1 | Bidirectional correlation between gastroesophageal reflux disease and sleep problems A systematic review and meta-analysis | L1-3 |
| **ABSTRACT** | | |  |
| Abstract | 2 | Objectives: The aim of this meta-analysis is to assess whether there is an association between sleep problems and gastroesophageal reflux disease.  Methods: PubMed, Cochrane Library, Embase and Web of Science were searched using Medical Subject Headings (MeSH) and keywords for articles published from the inception of the database until August 2023. All statistical analyses used Stata statistical software, version 14.0. A fixed effects model was used when p>0.1 and I^2^ ≤50%. If p<0.1 and I^2^ >50% indicated high heterogeneity, a random effects model was used. To assess publication bias, the funnel plot and Egger's test were used.  Results: This meta-analysis included 22 studies. The analysis shows that insomnia, sleep disturbance, or short sleep duration was associated with an increased risk of gastroesophageal reflux disease (OR =2.02, 95% CI: 1.64-2.49, p <0.001; I^2^ =66.4%; OR =1.98, 95% CI: 1.58-2.50, p <0.001, I^2^ =50.1%; OR =2.66, 95% CI: 2.02-3.15, p <0.001; I^2^ = 62.5%, respectively). Meanwhile, the gastroesophageal reflux disease was associated with an increased risk of poor sleep quality (OR =1.47, 95% CI: 1.47-1.79, p <0.001, I^2^ =72.4%), sleep disturbance (OR =1.47, 95% CI: 1.24-1.74, p <0.001, I^2^ = 71.6%), or short sleep duration (OR=1.17, 95% CI: 1.12-1.21, p <0.001, I^2^ =0).  Conclusion: This meta-analysis shows the bidirectional relationship between four different types of sleep problems and GERD. The results of our meta-analysis facilitate the development of new ideas for the treatment of GERD and sleep problems. | L7-25 |
| **INTRODUCTION** | | |  |
| Rationale | 3 | Although there have been many studies related to factors gastroesophageal reflux disease, the relationship between sleep problems and GERD has been rarely noted. Clinicians need to know more about the clinical symptoms associated with GERD to help them make an earlier diagnosis. | L27-34 |
| Objectives | 4 | Our meta-analysis synthesizes the existing evidence on the link between gastroesophageal reflux disease and sleep problems and shows that gastroesophageal reflux disease and sleep problems have a bidirectional relationship. The results of this study open up new ideas for the clinical management of gastroesophageal reflux disease and sleep problems. | L35-41 |
| **METHODS** | | |  |
| Eligibility criteria | 5 | We included case-control or cohort studies that evaluate the association between gastroesophageal reflux disease (GERD) and sleep problems. A diagnosis of GERD could be made clinically by any of the following: a. questionnaire; b. endoscopy. Sleep problems include sleep disturbance, short sleep duration, insomnia and poor sleep quality. In this study, sleep disturbance means people were found to be struggling to fall asleep, or waking up too early and not being able to get back to sleep. Criteria for insomnia include difficulty initiating or maintaining sleep, according to the Diagnostic and Statistical Manual of Mental Disorders, 4th edition (DSM-IV) or self-report. Short sleep duration was defined as sleeping less than 7 hours on average per night. The Pittsburgh Sleep Quality Index or a Likert scale containing the question: how do you rate your sleep quality is used to assess sleep quality. Poor sleep quality is considered to be present if the patient's Pittsburgh Sleep Quality Index is higher than normal or if the patient reports that sleep quality is poor.  The exclusion criteria included the following: 1) Studies did not provide an odds ratio (OR) estimate with 95% confidence interval (CI). 2) literature with the same data. 3) conference abstracts, study protocols, duplicate publications and studies without outcomes of interest. | L54-63 |
| Information sources | 6 | We retrieved publicly accessible studies up to August 2023 from PubMed, Cochrane Library, Embase and Web of Science. The language is restricted to English. | L48-49 |
| Search strategy | 7 | The search strategy was a combination of medical subject headings (Mesh) and text words. The keywords used for the search were ‘gastro-esophageal reflux’, ‘gastric acid reflux’, ‘gastric acid reflux disease’, ‘gastro-esophageal reflux disease’, ‘reflux disease, gastro-esophageal’ and ‘sleep*’. The search terms used for the retrieval of articles have been listed in Supplementary Table 3-6. | L49-52 |
| Selection process | 8 | The selection of studies was carried out by two reviewers (XLT, SSW). They independently screened the literature according to the inclusion and exclusion criteria. Duplicate and irrelevant articles were first excluded from the titles and abstracts. The full text of potentially eligible articles was then downloaded and read to identify all eligible studies. Any controversial articles had to be discussed before a decision on their inclusion was made. The selection process is presented in the Figure 1. | L64-66 |
| Data collection process | 9 | Two reviewers (XLT, FJW) independently extracted the following information from all articles selected for inclusion in the meta-analysis: name of first author, type and source of study design, nation, age of participants, sample size, type of sleep problem, adjustment confounders. | L73-75 |
| Data items | 10a | We collect about name of first author, type and source of study design, nation, age of participants, sample size, type of sleep problem, adjustment confounders in those articles. | L95-97 |
|  | 10b | Specific data elements are detailed in Tables 1 and 2. | L99-106 |
| Study risk of bias assessment | 11 | Risk of bias was assessed using the Newcastle-Ottawa Scale (NOS) by broadly categorizing studies as case-control or cohort studies. | L77-81 |
| Effect measures | 12 | The NOS tool assesses risk of bias by assigning a star for each response that meets the criteria. According to the instrument, each study can receive a maximum of nine stars: four for selection, two for comparability and three for outcome, with more stars indicating higher study quality. Ratings of 0-3, 4-6 and 7-9 were considered as indicating low, moderate and high quality, respectively. | L78-81 |
| Synthesis methods | 13a | According to NOS criteria, the quality score of cohort studies ranged from 5 to 9 (Table S1). The adjusted OR and 95% CI from each study were used to assess the association between GERD and sleep problems. The χ2 test and I2 values were used for the assessment of heterogeneity. A fixed effects model was used when p>0.1 and I2 ≤50%. If p<0.1 and I2 >50% indicated high heterogeneity, a random effects model was used. To check the robustness of the overall effects, the sensitivity analysis was performed by excluding one study each time and rerunning the analysis. Publication bias was confirmed by visual inspection of funnel plots and statistical assessment using Egger’s regression test. We performed several analyses based on GERD and each type of sleep problem. All statistical analyses were carried out using the Stata statistical software package, version 14.0 (Stata Corp, College Station, Texas). | L78-81 |
|  | 13b |  | L78-81 |
|  | 13c |  | L78-81 |
|  | 13d |  | L78-81 |
|  | 13e |  | L78-81 |
|  | 13f |  | L78-81 |
| Reporting bias assessment | 14 | The assessment is described in the Methods. | L83-89 |
| Certainty assessment | 15 | The assessment is described in the Methods. | L83-89 |
| **RESULTS** | | |  |
| Study selection | 16a | Of the 22 studies that were included, 14 were case-control studies and 8 were cohort studies. These studies cover the period from 2005 to 2023. | L93-97 |
|  | 16b | The reason of inclusion and exclusion is described in the flow diagram. | L97 |
| Study characteristics | 17 | Characteristic of studies are detailed in Tables 1 and 2. | L99-106 |
| Risk of bias in studies | 18 | The quality of studies is showed in Supplement Table 1. | L108-110 |
| Results of individual studies | 19 | The results are described in the part of result and Table 1 and 2. | L99-106 |
| Results of syntheses | 20a | The results are described in the part of result and figure 1 and 2. | L111-149 |
|  | 20b | The results are described in the part of result and figure 1 and 2. | L111-149 |
|  | 20c | The results are described in the part of result and figure 1 and 2. | L111-149 |
|  | 20d | The results are described in the part of result and figure 1 and 2. | L111-149 |
| Reporting biases | 21 | The biases are described in the part of result, figure 4 and Supplement Table 2. | L150-154 |
| Certainty of evidence | 22 | The certainty of evidence is described in the part of result. | L111-154 |
| **DISCUSSION** | | |  |
| Discussion | 23a | Provide a general interpretation of the results in the context of other evidence. | L156-162 |
|  | 23b | Discuss any limitations of the evidence included in the review. | L163-172 |
|  | 23c | Discuss any limitations of the review processes used. | L163-172 |
|  | 23d | Discuss implications of the results for practice, policy, and future research. | L173-204 |
| **OTHER INFORMATION** | | |  |
| Registration and protocol | 24a | The protocols have been pre-registered with the International Prospective Register of Systematic Reviews (PROSPERO) platform under the approval number: CRD42023452348. | L43-46 |
|  | 24b | Indicate where the review protocol can be accessed, or state that a protocol was not prepared. | NA |
|  | 24c | Describe and explain any amendments to information provided at registration or in the protocol. | NA |
| Support | 25 | There is non-financial support for the review. | NA |
| Competing interests | 26 | The authors report no conflicts of interest. | L225-228 |
| Availability of data, code and other materials | 27 | All data can be found in the manuscript and supplement materials. |  |

*From:*  Page MJ, McKenzie JE, Bossuyt PM, Boutron I, Hoffmann TC, Mulrow CD, et al. The PRISMA 2020 statement: an updated guideline for reporting systematic reviews. BMJ 2021;372:n71. doi: 10.1136/bmj.n71

For more information, visit: <http://www.prisma-statement.org/>
