# Supplementary material for: Bidirectional correlation between gastroesophageal reflux disease and sleep problems: a systematic review and meta-analysis
Source: PeerJ. 2024 Apr 16;12:e17202. doi: 10.7717/peerj.17202 (PMC11027907; doi:10.7717/peerj.17202)
Supplement: Supplemental Information 10 [file peerj-12-17202-s010.docx]

| Study | Year | Selection | Comparability | Outcome | Total |
| --- | --- | --- | --- | --- | --- |
| Case-Control Study(n=14) | | | | | |
| Mari-Ann Wallander, et al | 2007 | ** | * | ** | 5 |
| F. CREMONINI, et al | 2009 | **** | ** | ** | 8 |
| Jansson C，et al | 2009 | *** | ** | ** | 7 |
| Mei-Jyh Chen, et al | 2009 | *** | * | ** | 6 |
| Guillaume Cadiot, et al | 2011 | ** | * | ** | 5 |
| Anna Lindam, et al | 2012 | **** | ** | ** | 7 |
| Gawon Ju, et al | 2013 | *** | ** | ** | 7 |
| Masatsugu Okuyama, et al | 2017 | *** | * | ** | 6 |
| Ghasem Yadegarfar, et al | 2018 | *** | * | ** | 6 |
| Wei-Yi Lei, et al | 2018 | **** | * | ** | 7 |
| J. L. Horsley-Silva，et al | 2019 | ** | ** | ** | 6 |
| Min Kyung Hyun, et al | 2019 | **** | ** | ** | 8 |
| Shahid Ahmed, et al | 2020 | *** | * | * | 5 |
| Chiu-Hua Chang, et al | 2021 | * | * | *** | 5 |
| Cohort Study(n=8) | | | | | |
| Ronnie Fass, et al | 2005 | *** | * | * | 5 |
| Jihui Zhang, et al | 2012 | **** | ** | * | 7 |
| Kimihiko Murase, et al | 2014 | **** | * | ** | 7 |
| Jihui Zhang, et al | 2011 | **** | ** | * | 7 |
| Zi-Hong You, et al | 2015 | *** | ** | ** | 7 |
| Anna Lindam, et al | 2016 | **** | ** | *** | 9 |
| Ossur Ingi Emilsson, et al | 2022 | **** | ** | ** | 8 |
| Jane Ha, et al | 2023 | ** | ** | ** | 6 |
