# Supplementary material for: Bidirectional correlation between gastroesophageal reflux disease and sleep problems: a systematic review and meta-analysis
Source: PeerJ. 2024 Apr 16;12:e17202. doi: 10.7717/peerj.17202 (PMC11027907; doi:10.7717/peerj.17202)
Supplement: Supplemental Information 11 [file peerj-12-17202-s011.docx]

| Risk of GERD cause by sleep problem | | Risk of Sleep Problems cause by GERD | |
| --- | --- | --- | --- |
| Subgroup | *P value* | Subgroup | *P value* |
| Insomnia | 0.37 | Poor Sleep Quality | 0.95 |
| Sleep Disturbance | 0.83 | Sleep Disturbance | 0.68 |
| Short Sleep Duration | 0.99 | Short Sleep Duration | 0.53 |
