# Supplementary material for: Bidirectional correlation between gastroesophageal reflux disease and sleep problems: a systematic review and meta-analysis
Source: PeerJ. 2024 Apr 16;12:e17202. doi: 10.7717/peerj.17202 (PMC11027907; doi:10.7717/peerj.17202)
Supplement: Supplemental Information 12 [file peerj-12-17202-s012.docx]

| PubMed |  |  |
| --- | --- | --- |
| NO. | Query | Results |
| #1 | Search: "Gastroesophageal Reflux"[Mesh] Sort by: Most Recent | 29269 |
| #2 | Search: (((((((((((((((((Gastric Acid Reflux[Title/Abstract]) OR (Acid Reflux, Gastric[Title/Abstract])) OR (Reflux, Gastric Acid[Title/Abstract])) OR (Gastric Acid Reflux Disease[Title/Abstract])) OR (Gastro-Esophageal Reflux Disease[Title/Abstract])) OR (Gastro Esophageal Reflux Disease[Title/Abstract])) OR (Gastro-Esophageal Reflux Diseases[Title/Abstract])) OR (Reflux Disease, Gastro-Esophageal[Title/Abstract])) OR (Gastro-esophageal Reflux[Title/Abstract])) OR (Gastro esophageal Reflux[Title/Abstract])) OR (Reflux, Gastro-esophageal[Title/Abstract])) OR (Gastroesophageal Reflux Disease[Title/Abstract])) OR (GERD[Title/Abstract])) OR (Reflux, Gastroesophageal[Title/Abstract])) OR (Esophageal Reflux[Title/Abstract])) OR (Gastro-Esophageal Reflux[Title/Abstract])) OR (Gastro Esophageal Reflux[Title/Abstract])) OR (Reflux, Gastro-Esophageal[Title/Abstract]) Sort by: Most Recent | 22040 |
| #3 | Search: ("Gastroesophageal Reflux"[Mesh]) OR ((((((((((((((((((Gastric Acid Reflux[Title/Abstract]) OR (Acid Reflux, Gastric[Title/Abstract])) OR (Reflux, Gastric Acid[Title/Abstract])) OR (Gastric Acid Reflux Disease[Title/Abstract])) OR (Gastro-Esophageal Reflux Disease[Title/Abstract])) OR (Gastro Esophageal Reflux Disease[Title/Abstract])) OR (Gastro-Esophageal Reflux Diseases[Title/Abstract])) OR (Reflux Disease, Gastro-Esophageal[Title/Abstract])) OR (Gastro-esophageal Reflux[Title/Abstract])) OR (Gastro esophageal Reflux[Title/Abstract])) OR (Reflux, Gastro-esophageal[Title/Abstract])) OR (Gastroesophageal Reflux Disease[Title/Abstract])) OR (GERD[Title/Abstract])) OR (Reflux, Gastroesophageal[Title/Abstract])) OR (Esophageal Reflux[Title/Abstract])) OR (Gastro-Esophageal Reflux[Title/Abstract])) OR (Gastro Esophageal Reflux[Title/Abstract])) OR (Reflux, Gastro-Esophageal[Title/Abstract])) Sort by: Most Recent | 36875 |
| #4 | Search: "Sleep"[Mesh] Sort by: Most Recent | 97816 |
| #5 | Search: sleep*[Title/Abstract] Sort by: Most Recent | 233432 |
| #6 | Search: ("Sleep"[Mesh]) OR (sleep*[Title/Abstract]) Sort by: Most Recent | 251110 |
| #7 | Search: (("Gastroesophageal Reflux"[Mesh]) OR ((((((((((((((((((Gastric Acid Reflux[Title/Abstract]) OR (Acid Reflux, Gastric[Title/Abstract])) OR (Reflux, Gastric Acid[Title/Abstract])) OR (Gastric Acid Reflux Disease[Title/Abstract])) OR (Gastro-Esophageal Reflux Disease[Title/Abstract])) OR (Gastro Esophageal Reflux Disease[Title/Abstract])) OR (Gastro-Esophageal Reflux Diseases[Title/Abstract])) OR (Reflux Disease, Gastro-Esophageal[Title/Abstract])) OR (Gastro-esophageal Reflux[Title/Abstract])) OR (Gastro esophageal Reflux[Title/Abstract])) OR (Reflux, Gastro-esophageal[Title/Abstract])) OR (Gastroesophageal Reflux Disease[Title/Abstract])) OR (GERD[Title/Abstract])) OR (Reflux, Gastroesophageal[Title/Abstract])) OR (Esophageal Reflux[Title/Abstract])) OR (Gastro-Esophageal Reflux[Title/Abstract])) OR (Gastro Esophageal Reflux[Title/Abstract])) OR (Reflux, Gastro-Esophageal[Title/Abstract]))) AND (("Sleep"[Mesh]) OR (sleep*[Title/Abstract])) Sort by: Most Recent | 1427 |
