# Supplementary material for: Bidirectional correlation between gastroesophageal reflux disease and sleep problems: a systematic review and meta-analysis
Source: PeerJ. 2024 Apr 16;12:e17202. doi: 10.7717/peerj.17202 (PMC11027907; doi:10.7717/peerj.17202)
Supplement: Supplemental Information 13 [file peerj-12-17202-s013.docx]

| Embase |  |  |
| --- | --- | --- |
| NO. | Query | Results |
| #1 | 'Gastroesophageal reflux'/mj | 26098 |
| #2 | 'gastric acid reflux':ab,ti OR 'acid reflux, gastric':ab,ti OR 'reflux, gastric acid':ab,ti OR 'gastric acid reflux disease':ab,ti OR 'gastro-esophageal reflux disease':ab,ti OR 'gastro esophageal reflux disease':ab,ti OR 'gastro-esophageal reflux diseases':ab,ti OR 'reflux disease, gastro-esophageal':ab,ti OR 'gastro-oesophageal reflux':ab,ti OR 'gastro oesophageal reflux':ab,ti OR 'reflux, gastro-oesophageal':ab,ti OR 'gastroesophageal reflux disease':ab,ti OR 'gerd':ab,ti OR 'reflux, gastroesophageal':ab,ti OR 'esophageal reflux':ab,ti OR 'gastro-esophageal reflux':ab,ti OR 'gastro esophageal reflux':ab,ti OR 'reflux, gastro-esophageal':ab,ti | 37805 |
| #3 | #1 OR #2 | 47524 |
| #4 | 'sleep'/mj | 56291 |
| #5 | sleep*:ab,ti | 350265 |
| #6 | #4 OR #5 | 354628 |
| #7 | #3 AND #6 | 2265 |
