# Supplementary material for: Bidirectional correlation between gastroesophageal reflux disease and sleep problems: a systematic review and meta-analysis
Source: PeerJ. 2024 Apr 16;12:e17202. doi: 10.7717/peerj.17202 (PMC11027907; doi:10.7717/peerj.17202)
Supplement: Supplemental Information 14 [file peerj-12-17202-s014.docx]

| Cochrane |  |  |
| --- | --- | --- |
| NO. | Query | Results |
| #1 | MeSH descriptor: [Gastroesophageal Reflux] explode all trees | 2566 |
| #2 | (Reflux, Gastro-oesophageal):ab,ti,kw OR (Reflux, Gastro-Esophageal):ab,ti,kw OR (Reflux Disease, Gastro-Esophageal):ab,ti,kw OR (Gastro-Esophageal Reflux):ab,ti,kw OR (Gastro Esophageal Reflux Disease):ab,ti,kw OR (Reflux, Gastric Acid):ab,ti,kw OR (Gastric Acid Reflux Disease):ab,ti,kw OR (Acid Reflux, Gastric):ab,ti,kw OR (Gastro-oesophageal Reflux):ab,ti,kw OR (Gastro-Esophageal Reflux Disease):ab,ti,kw OR (Gastro oesophageal Reflux):ab,ti,kw OR (GERD):ab,ti,kw OR (Gastroesophageal Reflux Disease):ab,ti,kw OR (Reflux, Gastroesophageal):ab,ti,kw OR (Gastro-Esophageal Reflux Diseases):ab,ti,kw OR (Esophageal Reflux):ab,ti,kw OR (Gastric Acid Reflux):ab,ti,kw OR (Gastro Esophageal Reflux):ab,ti,kw | 5897 |
| #3 | #1 OR #2 | 5953 |
| #4 | MeSH descriptor: [Sleep] explode all trees | 9474 |
| #5 | (sleep*):ti,ab,kw | 51927 |
| #6 | #4 OR #5 | 52011 |
| #7 | #3 AND #6 | 296 |
