# Supplementary material for: Bidirectional correlation between gastroesophageal reflux disease and sleep problems: a systematic review and meta-analysis
Source: PeerJ. 2024 Apr 16;12:e17202. doi: 10.7717/peerj.17202 (PMC11027907; doi:10.7717/peerj.17202)
Supplement: Supplemental Information 15 [file peerj-12-17202-s015.docx]

| Web of science |  |  |
| --- | --- | --- |
| NO. | Query | Results |
| #1 | Gastroesophageal Reflux OR Gastric Acid Reflux OR Acid Reflux, Gastric OR Reflux, Gastric Acid OR Gastric Acid Reflux Disease OR Gastro-Esophageal Reflux Disease OR Gastro Esophageal Reflux Disease OR Gastro-Esophageal Reflux Diseases OR Reflux Disease, Gastro-Esophageal OR Gastro-oesophageal Reflux OR Gastro oesophageal Reflux OR Reflux, Gastro-oesophageal OR Gastroesophageal Reflux Disease OR GERD OR Reflux, Gastroesophageal OR Esophageal Reflux OR Gastro-Esophageal Reflux OR Gastro Esophageal Reflux OR Reflux, Gastro-Esophageal | 43596 |
| #2 | sleep* | 302567 |
| #3 | #1 AND #2 | 1974 |
