# Supplementary material for: Bidirectional correlation between gastroesophageal reflux disease and sleep problems: a systematic review and meta-analysis
Source: PeerJ. 2024 Apr 16;12:e17202. doi: 10.7717/peerj.17202 (PMC11027907; doi:10.7717/peerj.17202)
Supplement: Supplemental Information 9 [file peerj-12-17202-s009.docx]

Dear Editor(s),

Available for your review is our manuscript entitled “Bidirectional correlation between gastroesophageal reflux disease and sleep problems A systematic review and meta-analysis” submitted for publication in PeerJ.

The rationale for conducting the meta-analysis is that prevalence of gastroesophageal reflux disease is rising, urging deeper exploration. Enhancing clinician awareness of GERD-related symptoms is crucial for earlier diagnosis. Prior meta-analyses confirm higher GERD prevalence in obese, smoking, and NSAID-using individuals. Moreover, reflux links to apnea, reduced sleep efficiency, and lowered oxygen levels during sleep, highlighting an underexplored connection between sleep issues and GERD.

About the second question: the contribution that it makes to knowledge in light of previously published related reports, including other meta-analyses and systematic reviews.

We systematically reviewed population-based evidence to ascertain the two-way connection between GERD and sleep problems. The results of our meta-analysis facilitate the development of new ideas for the treatment of GERD and sleep problems.

We thank you for your time and consideration of this manuscript.

Sincerely,

Jun Zhu, M.D.
